# Release of adenosine from human neutrophils stimulated by platelet activating factor, leukotriene B_4_ and opsonized zymosan

**DOI:** 10.1155/S0962935192000413

**Published:** 1992

**Authors:** S. Sipka, Z. Dinya, M. Koltai, P. Braquet, G. Szegedi

**Affiliations:** 1 3rd Department of Internal Medicine University Medical School of Debrecen Debrecen Hungary; 2 Institute of Organic Chemistry Lajos Kossuth University Debrecen Hungary; 3 Institut Henri Beaufour Le Plessis Robinson France

## Abstract

Isolated human polymorphonuclear leukocytes (PMNL) stimulated by platelet activating factor (PAF), leukotriene B_4_ (LTB_4_) or opsonized zymosan (OZ) released adenosine measured by thermospray high performance liquid chromatography mass spectrometry in the cell-free supernatants. Stimulation by PAF or LTB_4_ resulted in a bellshaped concentration-effect curve; 5 × 10^−7^ M PAF, 10^−8^ M LTB_4_ and 500 μg ml^−1^ OZ induced peak adenosine release, thus cytotoxic concentrations did not elevate adenosine level in the supernatants. Therefore adenosine release was characteristic of viable cells. As calculated from concentration-effect curves, the rank order of potency for adenosine release was PAF > LTB > OZ. These resuits suggest that adenosine, when bound specifically to membrane receptor sites, may initiate signal transduction, and, in co-operation with other inflammatory mediators, may modulate phagocyte function, e.g. production of chemoluminescence (CL).


Mediators of Inflammation, 1, 267–271 (1992)

### Release of adenosine from human neutrophils stimulated by platelet activating factor, leukotriene $B_4$ and opsonized zymosan

S. Sipka,[1,CA] Z. Dinya,[2] M. Koltai,[3] P. Braquet[3] and G. Szegedi[1]

[1]3rd Department of Internal Medicine, University Medical School of Debrecen;
[2]Institute of Organic Chemistry, Lajos Kossuth University, Debrecen, Hungary;
[3]Institut Henri Beaufour, Le Plessis Robinson, France

[CA] Corresponding Author

**Isolated human polymorphonuclear leukocytes (PMNL) stimulated by platelet activating factor (PAF), leukotriene $B_4$ ($LTB_4$) or opsonized zymosan (OZ) released adenosine measured by thermospray high performance liquid chromatography mass spectrometry in the cell-free supernatants. Stimulation by PAF or $LTB_4$ resulted in a bell-shaped concentration-effect curve; $5 \times 10^{-7}$ M PAF, $10^{-8}$ M $LTB_4$ and 500 µg $ml^{-1}$ OZ induced peak adenosine release, thus cytotoxic concentrations did not elevate adenosine level in the supernatants. Therefore adenosine release was characteristic of viable cells. As calculated from concentration-effect curves, the rank order of potency for adenosine release was PAF > $LTB_4$ > OZ. These results suggest that adenosine, when bound specifically to membrane receptor sites, may initiate signal transduction, and, in co-operation with other inflammatory mediators, may modulate phagocyte function, e.g. production of chemoluminescence (CL).**

**Key words:** Adenosine, Chemoluminescence, $LTB_4$, Opsonized zymosan, PAF, Polymorphonuclear leukocytes

### Introduction

Platelet activating factor (PAF) is a phospholipid autacoid implicated as mediator in the pathogenesis of inflammation, thrombosis, immune disorders, septic shock and a great variety of physiological and pathophysiological conditions.[1,2] PAF as second messenger of diverse injurious stimuli releases eicosanoids and superoxide anions from leukocytes, macrophages and endothelial cells.[3]

Leukotrienes (LTs) are metabolites of arachidonic acid (AA) formed by 5′-lipoxygenase. One leukotriene, $LTB_4$, is a potent chemotactic and aggregating agent released from polymorphonuclear leukocytes (PMNL).[4,5] $LTB_4$ is also involved in a variety of pathophysiological processes, including $\gamma$-interferon production.[6]

Phagocytosis induced by opsonized zymosan (OZ) is one of the most widely used models for testing the function of stimulated PMNL.[7] Engulfment of particles via Fc, CR1 and CR3 receptors involves marked changes in cellular metabolism, leading to degranulation and production of superoxide anions.[8]

Adenosine is a natural nucleoside known to regulate various cellular functions, including neurotransmission[9] and local circulation.[10] These effects of adenosine appear to be mediated by two separate subtypes of binding sites, i.e., $A_1$ and $A_2$ receptors.[11] By interacting with one of these receptor subtypes, adenosine can initiate a transmembrane signal which then may inhibit or stimulate adenylate cyclase via $A_1$ or $A_2$ receptors, respectively. A third adenosine recognition site, termed the P site, located on the catalytic subunit of adenylate cyclase, is activated by relatively high concentrations of adenosine.[12] Near micromolar concentrations of adenosine, interacting with $A_2$ receptors, have been shown to inhibit PMNL functions.[13]

The present experiments were designed to study the production and release of adenosine from PMNL stimulated by PAF, $LTB_4$ and OZ. The results obtained suggest that adenosine released from PMNL may induce inhibitory action, which then, as a regulatory feedback signal, may protect the phagocytes from irreversible damage due to overstimulation during the inflammatory process.

### Materials and Methods

*Materials:* Acetonitrile (HPLC grade, Baker Chemicals, Düsseldorf, Germany), adenosine, bovine serum albumin (BSA), 2-chloroadenosine, luminol (5-amino-2,3-dihydro-1,4-phthalazinedione, Sigma Chemicals, St. Louis, MO), dextran (molecular mass 70 kDa, Macrodex) and zymosan (Mannozym, Human Serobacteriological Institute, Budapest, Hungary), Ficoll (Pharmacia Fine Chemicals, Uppsala, Sweden), $LTB_4$ dissolved in 99.5% ethanol (Calbiochem, Lucerne, Switzerland), PAF (Bachem, Bubendorf, Switzerland), TC-199 medium (Parker medium, Institute of Hygiene, Budapest, Hungary),

© 1992 Rapid Communications of Oxford Ltd trypan blue (Reanal, Budapest, Hungary), Uromiro (Bracco Chimica, Milan, Italy) were used, PAF was dissolved in phosphate-buffered saline (PBS), containing 1 mg $ml^{-1}$ of BSA.

*Separation of cells:* Heparinized venous blood was obtained from healthy donors. After removal of mononuclear cells by Ficoll-Uromiro density gradient centrifugation, the neutrophil-rich pellet was sedimented by dextran. Residual erythrocytes were lysed by hypotonic saline (0.45%). The final cell suspension consisted of 95% PMNL and 5% mononuclears but no red blood cells.[14]

*Measurement of adenosine:* A thermospray high performance liquid chromatography mass spectroscopy (TSP-HPLC-MS)[15,16] was used to determine adenosine concentrations in cell-free supernatants of PMNL stimulated with various concentrations of PAF, $LTB_4$ or OZ.[17,18] Proteins in 1 ml cell-free supernatants were precipitated by addition of acetonitrile (1:10, v/v) and subsequent centrifugation at 1500 ***g*** for 10 min. Aliquots of acetonitrile solution, 25 $\mu$l of each, were injected directly onto the HPLC column. HPLC was carried out using a Water C 18 Nova-Pak RP column (15 × 0.40 cm I.D.) on a Model 640 gradient controller and Model U6K injector. A linear gradient from 20.5% to 70% acetonitrile in 0.05 M aqueous ammonium acetate (1 ml $min^{-1}$) was used. The water for HPLC was Milli-Q quality. Sample ionization was achieved by thermospraying the HPLC eluent into a VG-TRIO-2 quadripole mass spectrometer (VG MassLab, UK) via a VG thermospray/plasmaspray interface. The ion source temperature was held at 200°C, TSP probe temperature was 210°C. Adenosine shows a base peak corresponding to the $[M^{+}H]^{+}$ ion at $m/_z$ 268. The mass spectrometer monitored the eluent continuously at $m/_z$ 268.

*Measurement of chemiluminescence:* 2 × $10^6$ PMNL were incubated in the presence of 5 × $10^{-7}$ M PAF, dissolved in PBS/BSA, and/or with 2-chloroadenosine in various experimental conditions at 37°C for 60 min. Chemiluminescence of PMNL was measured in PBS at a final volume of 1 ml in the presence of $10^{-7}$ M luminol using a Nuclear Chicago/300 liquid scintillation counter (Searle, Indianapolis, IN) in the off coincidence mode.[19,20]

*Viability of cells:* Viability was determined by the trypan-blue exclusion test to detect the percentage of viable cells at the beginning of the experiments and to check the cytotoxic effect of PAF, $LTB_4$ and OZ at the end of incubation with these stimulating agents.[21]

*Statistical analysis:* Data are expressed as means ± standard deviation (SD). Each adenosine determination was carried out in triplicate samples. Data were statistically analyzed by the tailed Student's *t*-test. Differences were considered significant when *p* was less than 0.05.

### Results

*Adenosine release:* Adenosine release was studied in samples of 2 × $10^6$ PMNL $ml^{-1}$ incubated with various concentrations of PAF, $LTB_4$ and OZ in TC-199 medium under 5% $CO_2$ for 30 and 60 min. Adenosine content in the supernatants was determined by TSP-HPLC-MS, and measured values were compared to that obtained in non-stimulated cell suspensions. In supernatants obtained from non-stimulated cells adenosine concentrations were below the limit of detectability. PAF at $10^{-8}$ M concentration resulted in a well established adenosine release; maximal production was induced by 5 × $10^{-7}$ M, whereas after exposure to $10^{-4}$ M no detectable adenosine concentrations were measured in the supernatants (Figure 1). The cytotoxic effect of $10^{-4}$ M PAF was 85%. This bell-shaped concentration-effect curve indicates that extracellular appearance of adenosine may be due to intracellular metabolic processes rather than cell damage.

$LTB_4$ also increased adenosine content of supernatants in a similar manner (Figure 2). Peak release was observed at $10^{-8}$ M. The highest concentration of adenosine released by $LTB_4$-stimulated PMNL remained, however, below the values that were produced by PAF. This corresponded to a lower cytotoxicity of $LTB_4$; the cytotoxic effect of $10^{-5}$ M $LTB_4$ was only 20%. The calculated ratio of adenosine production, i.e., total adenosine released by PAF/total adenosine released

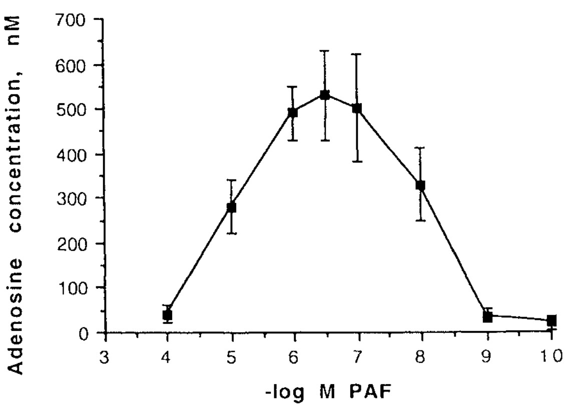

FIG. 1. Release of adenosine from human PMNL stimulated by various concentrations of PAF. 2 × $10^6$ PMNL $ml^{-1}$ were incubated in TC-199 medium at 37°C for 60 min. PAF was dissolved in PBS/BSA and 50 $\mu$l was added to each sample to reach its final concentration in 1 ml. Adenosine concentrations in cell-free supernatants were determined by TSP-HPLC-MS; means ± SD, $n = 3$.

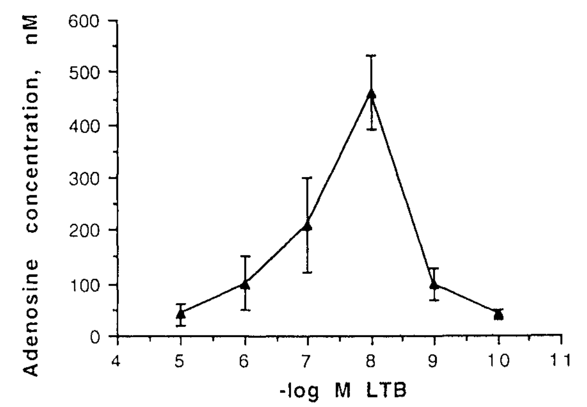

FIG. 2. Release of adenosine from human PMNL stimulated by various concentrations of $LTB_4$. 2 × $10^6$ PMNL $ml^{-1}$ were incubated in TC-199 medium at 37°C for 60 min. $LTB_4$ was dissolved in 99.5% ethanol then diluted with the incubation medium. The solvent itself, added in 3 μl to the 1 ml incubation medium, had no effect on adenosine release. Adenosine concentrations in cell-free supernatants were determined by TSP-HPLC-MS; means ± SD, $n = 3$.

by $LTB_4$, in the concentration ranges of $10^{-9}$ to $10^{-4}$ M or $10^{-10}$ to $10^{-5}$ M PAF or $LTB_4$ respectively was 2.25, indicating an efficacy for PAF higher than for $LTB_4$.

The kinetics of adenosine release were determined in PMNL stimulated with 5 × $10^{-7}$ M PAF, $10^{-8}$ M $LTB_4$ and 500 μg $ml^{-1}$ OZ at 37°C for 30 and 60 min. This entirely non-cytotoxic concentration of OZ was used since it was shown to induce the highest CL of PMNL.[22] As shown in Table 1, adenosine concentrations in the supernatants of cell suspensions stimulated for 30 min were lower than that measured after 60 min stimulation. Stimulation by OZ for 30 min did not produce any detectable elevation of adenosine concentration. The rank order of potency for adenosine release was PAF > $LTB_4$ > OZ.

*Chemiluminescence:* The inhibition by adenosine of CL in PMNL was demonstrated in transfer experiments. Supernatants of PMNL incubated in the presence of 5 × $10^{-7}$ M PAF, containing approximately 400 nM adenosine, were transferred to unstimulated cells, and the luminol induced amplification of CL in these cells was measured, or PAF induced CL was determined in the presence of 2-chloroadenosine, a stable analogue of adenosine. Preincubation of PMNL with PAF at 37°C for 60 min produced adenosine concentrations that were able to inhibit the CL of freshly added PAF when transferred to unstimulated cells. This stimulation was almost as intense as that was seen after addition of $10^{-6}$ M 2-chloroadenosine (Table 2).

**Table 1.** Adenosine concentrations in the supernatants of human PMNL stimulated by PAF, $LTB_4$ and opsonized zymosan

| Incubation, min | Concentration of adenosine, nM means ± SD — Stimulation by: PAF 5 × $10^{-7}$ M | $LTB_4$ $10^{-8}$ M | opsonized zymosan 500 μg $ml^{-1}$ |
|---|---|---|---|
| 30 | 405 ± 89 | 183 ± 81 | ND |
| 60 | 533 ± 151 | 351 ± 113 | 103 ± 41 |

2 × $10^6$ PMNL $ml^{-1}$ were incubated in TC-199 medium at 37°C. Adenosine concentrations in the cell-free supernatants were determined by TSP-HPLC-MS; $n = 3$, PMNL were used from three healthy donors, ND = not detectable.

**Table 2.** Effect of PAF and 2-chloroadenosine (2-c-Ado) on the chemiluminescence of human PMNL

| | Group | number of emitted photons, cpm means ± SD |
|---|---|---|
| 1 | PMNL in PBS/BSA 60 min (control) | 1152 ± 143 |
| 2 | PMNL in PBS/BSA 60 min plus PAF | 2114 ± 342* |
| 3 | PMNL plus PAF in PBS/BSA, 60 min | 1320 ± 211 |
| 4 | PMNL plus supernatant (3) plus PAF | 1457 ± 256 |
| 5 | PMNL in PBS/BSA 60 min plus 2-c-Ado | 1085 ± 117 |
| 6 | PMNL in PBS/BSA 60 min plus 2-c-Ado and PAF | 1188 ± 196 |

2 × $10^6$ PMNL were incubated at 37°C. PAF dissolved in PBS/BSA and 2-chloro-adenosine (2-c-Ado) were added at final concentrations of 5 × $10^{-7}$ M and $10^{-6}$ M, respectively. In transfer experiments the cell-free supernatant of PAF stimulated (adenosine containing) PMNL (sample 3) was added to freshly prepared, unstimulated cells (sample 4). In sample 6, PAF was added to the cells after preincubation of PMNL with 2-c-Ado for 10 min. Chemiluminescence, measured in a scintillation counter by the off coincidence mode, was induced by 5 × $10^{-7}$ M PAF added to the cells at the end of incubation, except when the background chemiluminescence of PMNL incubated alone (sample 1) or in the presence of 2-c-Ado (sample 5) was determined; $n = 4$, PMNL of the same healthy donors were used in four separate experiments. *$p < 0.01$ as compared to control.

### Discussion

Beyond the lipid character, common features in the effects of PAF and $LTB_4$ are the induction of chemotaxis, aggregation and superoxide anion production in PMNL.[1,23,24] This study shows that both PAF and $LTB_4$ release adenosine from PMNL, although the effect of PAF is more marked than that of $LTB_4$. The difference may be explained by the fact that PAF can also release $LTB_4$,[3,18] thus adenosine release induced by PAF presumably includes that released by $LTB_4$. Regardless of distinct receptor binding sites for PAF and $LTB_4$ on PMNL membrane,[6,24-26] these findings point to the similarity of signal transduction triggered by the two autacoids, suggesting their involvement in a common pathway of the inflammatory process.

The particles of OZ are internalized by PMNL via Fc, CR1 and CR3 receptors leading to degranulation of specific azurophil granules and production of superoxide anion.[27] In our study, OZ proved to be the least effective in releasing adenosine from human PMNL. Stimulated PMNL produce PAF and $LTB_4$ at picomolar concentrations,[28] and this may explain the finding that adenosine concentrations are much lower in supernatants of cells stimulated with OZ than in supernatants stimulated with higher, micromolar concentrations of exogenous PAF or $LTB_4$. This can also be reflected by the kinetics of adenosine production in stimulated cells. While 30 min stimulation was found to be optimal for production of adenosine by PAF or $LTB_4$, this incubation period was insufficient in OZ-activated PMNL[17,29] to detect a measurable amount of adenosine in the supernatant, while 60 min stimulation resulted in a well established increase of adenosine concentration. Accordingly, adenosine production by PAF and $LTB_4$ was also more marked after 60 min than at 30 min stimulation.

The preparation of a completely platelet free human PMNL suspension is practically impossible. The rate of platelet contamination in our PMNL suspensions was ordinarily 1:1. The estimated amount of adenosine possibly derived from aggregated platelets was 50 nM. To aggregate platelets but not neutrophils, ADP was added to cells suspended in TC-199 medium, and adenosine release was measured in the supernatants 60 min later (data not shown). From these results the conclusion can be drawn that the major part of adenosine released by PAF, $LTB_4$ and OZ is derived from PMNL.

Adenosine is produced by the breakdown of intracellular ATP, and an increased consumption of ATP results from the stimulation of phagocytes via the pathway of ATP synthesis from ADP:

$$2\text{ADP} \longrightarrow \text{ATP} + \text{AMP} \quad (1)$$

$$\text{AMP} \xrightarrow{5'\text{-nucleosidase}} \text{adenosine}^{22} \quad (2)$$

We assume that, at a certain degree of ATP depletion in activated cells, this process may lead to accumulation of adenosine at nearly micromolar concentrations in the extracellular space, because some adenosine molecules may escape from the rapid breakdown by adenosine deaminase located on the external surface of cells.[30] These molecules may then bring important signal transduction for regulating the function of surrounding cells. Adenosine binding $A_2$ receptor subtypes and P sites, has been shown to inhibit PMNL functions, e.g. intracellular killing or generation of oxygen derived free radicals.[31] As previously described,[11] this effect is related to an activation of adenylate cyclase with a concomitant elevation of intracellular cAMP level. Intracellular cAMP raised by eicosanoids, in particular prostacyclin ($PGI_2$), has been shown to downregulate eicosanoid production in platelets[32,33] and vascular endothelium.[34] PAF has been shown to release AA by conversion not only to the lipoxygenase product $LTB_4$,[3,18] but also through the cyclooxygenase pathway to prostaglandin $E_2$ ($PGE_2$)[35] and $PGI_2$.[36] All these events have been shown to augment the number of emitted photons in luminol induced CL.[37] Eicosanoid synthesis by itself is therefore associated with increased light emission[38] and indomethacin can block such CL.[39] Consequently, the elevation of intracellular cAMP by PGs, the endproducts of AA metabolism, is accompanied by decreased CL as a consequence of feedback inhibition brought about either directly as a result of decreased precursor conversion or through the NADPH oxidase system.[40] Thus, CL of phagocytes can be modulated not only by adenosine released from stimulated cells, but also by AA and its biologically highly active endproducts depending upon the stage of their production. At the same time, a great variety of interactions may occur among inflammatory mediators, for example, adenosine can block LT synthesis.[41] An elevated intracellular cAMP level, induced by either adenosine,[42] $PGE_2$[41] or PAF[43] may be an important signal transduction to downregulate CL,[44] thus being a sensitive marker for the metabolic and functional state of phagocytes. Another important aspect of this autoregulatory feedback network is that cAMP has also been implicated in the inhibition of PAF release.[45] On the other hand, the extracellular nucleotides, such as ATP, ADP and AMP, may induce the opposite effect, i.e., stimulation of adhesiveness and other functions of PMNL.[46]

This regulatory and inhibitory role for exogenously applied and endogenously released adenosine has been described in stimulated PMNL.[47–49] Our data confirm these observations and support the view that production and release of adenosine may regulate the function of activated PMNL and other phagocytes. In the autocatalytic feedback network of inflammatory mediator release,[50] adenosine may therefore be regarded as an important signal molecule downregulating the production of PAF, $LTB_4$ and other lipid mediators, and through this mechanism activated PMNL and other phagocytes may be protected from potentially irreversible damage due to overstimulation.[51,52] This modulatory action, however, takes place in co-operation with other mediators, mainly AA derivatives.

### References

1. Braquet P, Tuqui L, Shen TY, Vargaftig BB. Perspectives in platelet activating factor research. *Pharmacol Rev* 1987; **39**: 97–145.
2. Koltai M, Hosford D, Guinot P, Esanu A, Braquet P. Platelet-activating factor (PAF): a review of its effects, antagonists and possible future clinical applications. *Drugs* 1991; **42:I** 9–29, **II** 174–204.

3. Stewart AG, Dubbin PN, Harris T, Dusting GJ. Platelet-activating factor may act as a second messenger in the release of eicosanoids and superoxide anions from leukocytes and endothelial cells. *Proc Natl Acad Sci USA* 1990; **87**: 3215–3219.
4. Ford-Hutchinson AW, Bray MA, Doing MV, Shipley MK, Smith MJH. Leukotriene B: a potent chemotactic and aggregating substance released from polymorphonuclear leucocytes. *Nature* 1980; **286**: 264–265.
5. Lin AH, Ruppel PL, Gorman, RR. Leukotriene $B_4$ binding to human neutrophils *Prostaglandins* 1984; **28**: 873–883.
6. Johnson HM, Torres BA. Leukotrienes: positive signals for regulating $\gamma$-interferon production. *J Immunol* 1984; **132**: 413–417.
7. Cline JM, Territo MC. Phagocytosis. In: Parker CL ed. *Clinical Immunology I*, Philadelphia, London, Toronto: Saunders 1980; 296–313.
8. Hurst NP. Molecular basis of activation and regulation of the respiratory burst. *Ann Rheum Dis* 1987; **46**: 256–272.
9. Kulkarni SK, Thorat SN. Purinergic neurotransmission: an update. *Drugs Today* 1990; **26**: 499–523.
10. Berne RM, Knabb RM, Ely SW, Rubio R. Adenosine in the local regulation of blood flow: a brief review. *Fed Proc* 1983; **42**: 3136–3142.
11. Londos C, Wolff J. Two distinct adenosine-sensitive sites on adenylate cyclase. *Proc Natl Acad Sci USA* 1977; **74**: 5482–5486.
12. Haslam RJ, Davidson MML, Desjardins TV. Inhibition of adenylate cyclase by adenosine analogues in preparations of broken and intact human platelets. Evidence for the undirectional control of platelet function by cyclic AMP. *Biochem J* 1978; **176**: 83–95.
13. Marone G, Petracca R, Vigorita S. Adenosine receptors on human inflammatory cells. *Int Archs Allergy Appl Immunol* 1985; **77**: 259–263.
14. Böyum A. Separation of leukocytes from blood and bone marrow. *Scand J Clin Invest* 1968; **21(Suppl. 97)**: 9–109.
15. Yergey AL, Edmonds CG, Lewis IAS, Vestal ML. In: Hercules D, ed. *Liquid Chromatography/Mass Spectrometry: Techniques and Applications.* New York: Plenum Press, 1990; 105–125.
16. Games DE. Combined high-performance liquid chromatography mass spectrometry. *Anal Proc* London, 1980; **17**: 110–116.
17. Fradin A, Zirolli JA, Maclouf J, *et al.* Platelet-activating factor and leukotriene biosynthesis in whole blood. *J Immunol* 1989; **143**: 3680–3685.
18. McColl SR, Krump E, Naccache PH, *et al.* Granulocyte-macrophage colony-stimulating factor increases the synthesis of leukotriene $B_4$ by human neutrophils in response to platelet-activating factor. Enhancement of both arachidonic acid availability and 5-lipoxygenase activation. *J Immunol* 1991; **146**: 1204–1211.
19. Sipka S, Abel G, Csongor J, Nyirkos. P, Fachet J. Effect of Mannozyn on the chemiluminescence of phagocytes. *Acta Microbiol Hung* 1986; **33**: 263–270.
20. Sipka S, Szentmiklosi J, Nagy A, Taskov V, Szegedi G. Inhibition of the zymosan induced chemiluminescence of human phagocytes by adenosine, polyadenylic acid and agents influencing adenosine metabolism. *Allergol Immunol* 1989; **17**: 209–212.
21. Batchelor JR. Assays for cytotoxic and haemagglutinating antibodies against histocompatibility antigens. In: Weis DM ed. *Handbook of Experimental Immunology*, Oxford, Edinburgh: Blackwell, 1967; 999.
22. Harris RA. Carbohydrate metabolism I. Major metabolic pathways and their control. In: Devlin TM ed. *Textbook of Biochemistry with Clinical Correlations.* Second Edition, New York, Rochester, Brisbane, Toronto, Singapore: John Wiley & Sons, 1986; 261–328.
23. O'Flaherty JT, Surles JR, Redman J, *et al.* Binding and metabolism of platelet-activating factor by human neutrophils. *J Clin Invest* 1986; **78**: 381–388.
24. Serhan CN, Radin A, Smolen JE, *et al.* Leukotriene $B_4$ is a complete secretagogue in human neutrophils: a kinetic analysis. *Biochem Biophys Res Commun* 1983; **107**: 1006–1012.
25. Di Marzo V, Galadari SHJ, Tippins JR, Morris HR. Interactions between second messengers: cyclic AMP and phospholipase $A_2$- and phospholipase C-metabolites. *Life Sci* 1991; **49**: 247–259.
26. Sha'afi Ri, Kolski TFP. Activation of the neutrophil. *Prog Allergy* 1988; **42**: 1–64.
27. Korchak HM. Polymorphonuclear cells in inflammation: signal transduction in neutrophils. In: Marone G, Lichtenstein LM, Condorelli M, Fauci AS eds. *Human Inflammatory Diseases. I.* Toronto, Philadelphia: Decker Inc, 1988; 255–262.
28. Cluzel M, Undem BJ, Chilton FH. Release of platelet-activating factor and the metabolism of leukotriene $B_4$ by human neutrophil when studied in a cell superfusion model. *J Immunol* 1989; **143**: 3659–3665.
29. Riches DWH, Young SK, Seccombe JP, *et al.* The subcellular distribution of platelet-activating factor in stimulated human neutrophils. *J Immunol* 1990; **145**: 3062–3070.
30. Aran JM, Colomer D, Matutes E, Vives-Corrons JL, Franco R. Presence of adenosine desaminase on mononuclear blood cells: immunological localization using light and electron microscopy. *J Histochem Cytochem* 1991; **39**: 1001–1008.
31. Skubitz KM, Wickharm NW, Hammerschmidt DE. Endogenous and exogenous adenosine inhibit granulocyte aggregation without altering the associated rise in intracellular calcium concentration. *Blood* 1988; **72**: 29–33.
32. Gorman RR, Bunting S, Miller OV. Modulation of human platelet adenylate cyclase by prostacyclin (PGX) *Prostaglandins* 1977; **13**: 377–388.
33. Tateson JE, Moncada S, Vane JR. Effects of prostacyclin (PGX) on cyclic AMP concentrations in human platelets. *Prostaglandins* 1977; **13**: 389–397.
34. Abigail F, Brotherton A, Houck JC. Role of $Ca^{2+}$ and cyclic AMP in the regulation of the production of prostacyclin by the vascular endothelium. *Proc Natl Acad Sci USA* 1982; **79**: 495–499.
35. Dahl ML. Aggregation and prostanoid releasing effects of platelet-activating factor and leukotrienes on human polymorphonuclear leukocytes and platelets. *Int Archs Allergy Appl Immunol* 1985; **76**: 145–150.
36. Zimmerman GA, McIntyre TM, Prescott SM. Production of platelet-activating factor by human endothelial cells: evidence of a requirement for specific agonists and modulation by prostacyclin. *Circulation* 1985; **72**: 718–727.
37. Cheung K, Archibald AC, Robinson MF. The original of chemoluminescence produced by neutrophils stimulated by opsonized zymosan. *J Immunol* 1983; **130**: 2324–2329.
38. Marnett LJ, Wlodawer P, Samuelson B. Light emission during the action of prostaglandin synthetase. *Biochem Biophys Res Commun* 1974; **60**: 1786–1794.
39. Wörner P. Arachidonic acid-stimulated platelet chemoluminescence. In: Van Dyke K, Castranova V eds. *Cellular chemoluminescence. Vol 3*, Boca Raton, FL: CRC Press Inc, 1987; 77–90.
40. Weissmann G, Korchak HD, Perez HD, Smolen JE, Goldstein IM, Hoffstein ST. The secretory code of the neutrophils. *J Reticuloendothel Soc* 1979; **26**: 687–700.
41. Hillyard PA, Nials AT, Skidmore IF, Vardey CJ. Characterization of the adenosine receptor responsible for the inhibition of histamine and SRS-A release from human lung fragments. *Br J Pharmacol* 1984; **83**: 337–345.
42. Cronstein BN, Levin RJ, Belanoff J, Weissmann G, Hirschhorn R. Adenosine: an endogenous inhibitor of neutrophil-mediated injury to endothelial cells. *J Clin Invest* 1986; **78**: 760–770.
43. Adolfs MJD, Beusengerg FD, Bonta IL. PAF-acether modifies human peritoneal macrophage cAMP levels in a biphasic fashion. *Agents Actions* 1989; **26**: 119–120.
44. Cronstein BN, Daguma L, Nichols D, Hutchinson AJ, Williams M. The adenosine/neutrophil paradox resolved: human neutrophils possess both $A_1$ and $A_2$ receptors that promote chemothaxis and inhibit $O_2^-$ generation, respectively. *J Clin Invest* 1990; **85**: 1150–1157.
45. Heller R, Bussolino F, Chido D, *et al.* Protein kinase C and cyclic AMP modulate thrombin-induced platelet-activating factor synthesis in human endothelial cells. *Biochim Biophys Acta* 1991; **1093**: 55–64.
46. Freyer DR, Boxer LA, Axtell RA, Todd RF. Stimulation of human neutrophil adhesive properties by adenosine nucleotides. *J Immunol* 1988; **141**: 580–586.
47. Iannone MK, Zimmermann TP, Reynolds-Vaughn R, Wolberg G. Effects of adenosine on human neutrophil function and cyclic AMP content. In: Gerlach E, Becker BF eds. *Topics and Perspectives in Adenosine Research.* Berlin: Springer, 1987; 286–297.
48. Iannone MA, Wolber G. Effects of adenosine on neutrophil polarization induced by N-formyl-methionyl-leucyl-phenylalanine, sodium propionate and colchicine. *Agents Actions* 1989; **27**: 403–406.
49. Iannone MA, Wolber G, Zimmermann TP. Chemotactic peptide induces cAMP elevation in human neutrophils by amplification of the adenylate response to endogenously produced adenosine. *J Biol Chem* 1989; **264**: 20177–20180.
50. Braquet P, Paubert-Braquet M, Bourgain R, Bussolino F, Hosford, D. PAF/cytokine autogenerated feedback networks in microvascular immune injury: consequences in shock, ischemia and graft rejection. *J Lipid Med* 1990; **1**: 75–112.
51. Dewald B, Braggilioni M. Activation of NADPH oxidase in human neutrophil products PAF and $LTB_4$. *Biochem Biophys Res Commun* 1985; **128**: 297–304.
52. Doebber TW, Wu MS. Platelet-activating factor (PAF) stimulates the PAF synthesizing enzyme acetyl CoA: 1-alkyl-sn-glycero-3-phosphocholine $O_2$-acetyltransferase and PAF synthesis in neutrophils. *Proc Natl Acad Sci USA* 1987; **84**: 7557–7561.

ACKNOWLEDGEMENTS. This work was supported by the Hungarian Ministry of Health and Social Welfare and the Hungarian National Foundation for Scientific Research. We are grateful to Ms Ildiko Kovacs for her excellent technical assistance, and to Dr A. Gecse for providing a sample of $LTB_4$.

**Received 4 April 1992; accepted in revised form 12 June 1992**
